# The Role of Whey in Functional Microorganism Growth and Metabolite Generation: A Biotechnological Perspective

**DOI:** 10.3390/foods14091488

**Published:** 2025-04-24

**Authors:** Iuliu Gabriel Malos, Andra-Ionela Ghizdareanu, Livia Vidu, Catalin Bogdan Matei, Diana Pasarin

**Affiliations:** 1 Faculty of Animal Productions Engineering and Management, University of Agronomic Sciences and Veterinary Medicine of Bucharest, 59 Marasti Blvd., District 1, 011464 Bucharest, Romania; gabriel-iuliu.malos@usamv.ro (I.G.M.);; 2 National Research and Development Institute for Chemistry and Petrochemistry—ICECHIM, 202 Splaiul Independentei, 060021 Bucharest, Romania

**Keywords:** whey valorization, functional microorganisms, bacteriocins, sustainable bioprocessing fermentation process

## Abstract

The valorization of cheese whey, a rich by-product of the dairy industry that is rich in lactose (approx. 70%), proteins (14%), and minerals (9%), represents a promising approach for microbial fermentation. With global whey production exceeding 200 million tons annually, the high biochemical oxygen demand underlines the important need for sustainable processing alternatives. This review explores the biotechnological potential of whey as a fermentation medium by examining its chemical composition, microbial interactions, and ability to support the synthesis of valuable metabolites. Functional microorganisms such as lactic acid bacteria (*Lactobacillus helveticus*, *L. acidophilus*), yeasts (*Kluyveromyces marxianus*), actinobacteria, and filamentous fungi (*Aspergillus oryzae*) have demonstrated the ability to efficiently convert whey into a wide range of bioactive compounds, including organic acids, exopolysaccharides (EPSs), bacteriocins, enzymes, and peptides. To enhance microbial growth and metabolite production, whey fermentation can be carried out using various techniques, including batch, fed-batch, continuous and immobilized cell fermentation, and membrane bioreactors. These bioprocessing methods improve substrate utilization and metabolite yields, contributing to the efficient utilization of whey. These bioactive compounds have diverse applications in food, pharmaceuticals, agriculture, and biofuels and strengthen the role of whey as a sustainable biotechnological resource. Patents and clinical studies confirm the diverse bioactivities of whey-derived metabolites and their industrial potential. Whey peptides provide antihypertensive, antioxidant, immunomodulatory, and antimicrobial benefits, while bacteriocins and EPSs act as natural preservatives in foods and pharmaceuticals. Also, organic acids such as lactic acid and propionic acid act as biopreservatives that improve food safety and provide health-promoting formulations. These results emphasize whey’s significant industrial relevance as a sustainable, cost-efficient substrate for the production of high-quality bioactive compounds in the food, pharmaceutical, agricultural, and bioenergy sectors.

## 1. Introduction

Cheese whey, the most common by-product of the dairy industry, represents both a major environmental challenge and a valuable biotechnological opportunity. Worldwide whey production exceeds 200 million tons per year, with Europe contributing around 52.2 million tons in 2023. Due to its high lactose content and high organic load, whey disposal generates serious environmental concerns, as it leads to water pollution, oxygen depletion, and eutrophication. These effects are reflected in a high biochemical oxygen demand (BOD, 30–50 g/L) and chemical oxygen demand (COD, 60–80 g/L) and make untreated whey a major pollutant. In this context, the biotechnological valorization of whey as a fermentation substrate for functional microorganisms offers a promising alternative for waste mitigation and the sustainable production of high-quality metabolites [1,2,3]. In light of the current environmental challenges, there has been a marked increase in the demand for stringent environmental regulations, which, in turn, elevates the necessity for substantial investments in waste treatment facilities. Due to its typical composition of whey dry matter, which includes around 70% lactose, 14% proteins, 9% minerals, 4% fats, and 3% lactic acid, whey has great potential for valorization [4]. The potential of whey as an ideal substrate for the cultivation of functional microorganisms and the extraction of valuable metabolites is increasingly recognized in the literature [5,6]. Whey offers numerous advantages for product development, such as a favorable nutritional profile that significantly supports microbial cell growth. This property, together with its cost-effectiveness and availability, makes whey an attractive resource for biotechnological applications [7,8].

Functional microorganisms cultivated on whey can produce a wide range of valuable metabolites, including enzymes such as proteases and β-galactosidase; organic acids such as lactic, acetic, propionic, and butyric acids; and EPSs with immunomodulatory properties, such as heteropolysaccharides (HePSs), and cholesterol-lowering properties. Also, whey fermentation enables the synthesis of antimicrobial compounds such as bacteriocins (e.g., nisin, lacticin) and bioactive peptides with antihypertensive, antioxidant, and antimicrobial effects. These metabolites are used in the food industry as preservatives and functional ingredients, in the pharmaceutical industry for therapeutic formulations, and in agriculture as biopreservatives and growth-promoting agents [9,10,11,12].

This review aims to explore the potential of whey as a substrate for the cultivation of functional microorganisms and the production of metabolites by analyzing its chemical composition, the fermentation processes, and the applications of the resulting metabolites. In this way, the importance of whey for the development of innovative biotechnologies is emphasized and a contribution is made to the creation of sustainable solutions for the use of industrial resources. The literature included was selected according to its relevance to the biotechnological valorization of whey, with a focus on functional microorganisms, fermentation techniques, and the production of bioactive metabolites.

## 2. Chemical Composition of Whey

Whey is the liquid by-product of curd formation during cheese production or casein coagulation. Whey, a nutrient-rich by-product of dairy processing, contains around 50–55% of the nutrients in milk. Its composition is complex and includes a variety of macronutrients, micronutrients, and bioactive compounds that make it a valuable resource for various industries.

Whey can be divided into two types based on the coagulation process of the milk. Sweet whey (pH value of 6.0–7.0) is produced during the manufacture of rennet casein or cheese with rennet as a coagulant. Sweet whey is usually associated with the production of hard and semi-hard cheeses. Studies in the literature indicate that the chemical composition of sweet whey is as follows: protein (6–10 g/L), milk fat (0.20–0.50 g/L), lactose (46–52 g/L), minerals (2.5–4.7 g/L), and lactic acid (2,6 g/L). Acid whey (pH value below 5.0) is produced during the manufacture of acid casein or cheese with acid as a coagulant. Acid whey is a by-product of fresh cheese or yogurt production. According to the research, the chemical composition of acid whey is characterized by the following values: protein (6–8 g/L), milk fat (0.3 g/L), lactose (44–46 g/L), minerals (4.3–7.2 g/L), and lactic acid (2.6 g/L) [13,14].

The most important cheeses that produce sweet whey are cheddar, Swiss, Mozzarella, and Munster [15]. Most cheese varieties that produce acid whey are soft white (cottage) cheese, cream cheese, and ricotta [16].

The differences in the nutritional properties of cheese whey depend on the type of cheese and the processing. Cheese whey from sweet curd is richer in protein and has a lower fat content than cheese whey from sour curd. Cheeses made from whole milk are richer in fat and protein than cheeses made from semi-skimmed or skimmed milk. The protein content of cheese whey made from the same milk base decreases as the rennet casein yield increases. Acid whey is often considered underutilized waste compared with sweet whey, as it contains lower protein concentrations [17].

Whey is processed into cheese, whey protein concentrate (WPC, protein content 25–89%), whey protein isolate (WPI, protein content 90–95%) or whey protein hydrolysate (WPH, protein content up to 80%), delactosed whey powder (protein content up to 30%), demineralized whey powder (protein content 15–35%), or demineralized–delactosed whey powder (protein content 15–35%) [18,19]. Due to its nutritional and functional properties, whey is increasingly being processed into high-quality products such as WPC and WPI. WPC is often used in sports nutrition, infant formula, and bakery products due to its balanced protein content and emulsifying properties. WPH, rich in peptides and easily digestible, is preferred in clinical nutrition and hypoallergenic formulas as well as in performance-enhancing beverages. Delactosed and demineralized whey powder is used as a cost-effective ingredient in confectionery, bakery products, and animal feed when the lactose or mineral content needs to be reduced. Due to its high protein purity and excellent functional properties, WPI has significant industrial value, particularly as a base material for the development of micro- and nanomaterials, including nanocapsules and biodegradable films, which are being researched for applications in sustainable agriculture and active packaging [20,21]. The growing demand for whey proteins has resulted in around 40% of processed whey solids being used in products such as whey protein concentrate, whey protein isolate, lactose, and permeate [22,23,24]. Whey can also be categorized according to the type of milk used to make the cheese, such as cow whey, goat whey, sheep whey, buffalo whey, and whey from other milk sources. Whey of different origins differs in its chemical composition, and therefore, also in its use. Bovine whey is the most commonly used whey in the food industry. In general, cheese is made from pasteurized milk that is free from pathogenic microorganisms and active spoilage microorganisms. The cheese whey processing industry mainly uses cheese made from pasteurized milk [25,26].

### 2.1. Protein Fraction

**Figure 1:**
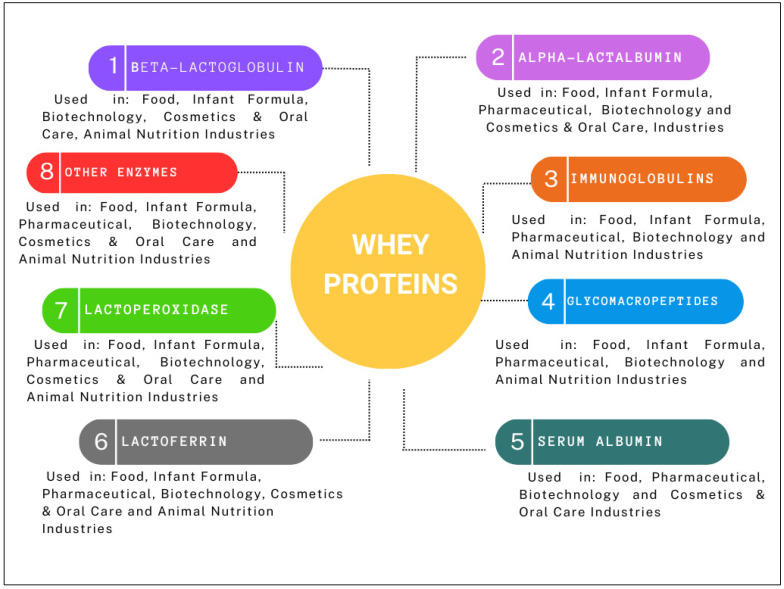
Whey protein types and their applications in various industries.

Whey proteins make up about 20% of the total protein content in milk. The most abundant whey proteins, listed in decreasing order of concentration, include β-lactoglobulin, α-lactalbumin, immunoglobulins, glycomacropeptide, serum albumin, lactoferrin, lactoperoxidase, and about 60 distinct enzymes (Figure 1) [27,28].

*β-lactoglobulin* is the most abundant whey protein (around 50% of the total whey protein). It is a water-soluble molecule (18.20–18.36 kDa) with the ability to bind and transport hydrophobic compounds such as lipids and fat-soluble vitamins.

Microorganisms can interact with β-lactoglobulin in different ways, either by adhesion mechanisms that allow them to bind to this protein [28] or by specific growth-promotion effects that enhance the nutritional value of the protein for the microorganism. Microorganisms adhere to proteins via mechanisms such as hydrophobic interactions, ionic attraction, hydrogen bonding, and van der Waals forces. In a soluble state, such as in whey solutions, the hydrophobicity of β-lactoglobulin facilitates adhesion due to the typically hydrophobic nature of microbial surfaces [29]. The presence of free (unfolded) β-lactoglobulin enhances the adhesion of certain microbial strains, while others may depend on specific proteins to adhere. Many microorganisms are able to hydrolyze β-lactoglobulin to promote their growth, although the exact mechanisms underlying these stimulatory effects are only partially understood.

The growth-promoting effect of microorganisms was investigated with β-lactoglobulin. As a reference experiment, the microbial growth profiles without β-lactoglobulin were first evaluated for five live species. After the growth of each species was confirmed, the growth with the addition of β-lactoglobulin was analyzed. In each case, the growth profile was significantly improved by the presence of β-lactoglobulin. Bacteria, yeasts, and filamentous fungi all showed an improved growth profile with the addition of β-lactoglobulin [30].

β-lactoglobulin can inhibit the growth of certain pathogens such as *Staphylococcus aureus* and *Streptococcus uberis*, mastitis-causing bacteria, but not *Escherichia coli*. Activity is concentration-dependent, specific for intact β-lactoglobulin, and varies between genetic variants, with β-lactoglobulin A showing greater efficacy than β-lactoglobulin B. Co-incubation of β-lactoglobulin with lactoferrin enhances the antibacterial effect, suggesting a complementary role in the defense of the mammary gland. β-lactoglobulin and lactoferrin contribute synergistically to the defense against pathogens, with β-lactoglobulin providing selective antimicrobial effects based on bacterial species [31].

Another study showed that sixty-four LAB strains cultured for 24 h at 37 °C in 10% and 16% (*w*/*v*) reconstituted whey powder degraded whey proteins to varying degrees, targeting β-lactoglobulin in particular, with the highest degradation rates (16–18%) recorded for *Lactobacillus acidophilus* CRL 636 and *Lactobacillus delbrueckii* subsp. *bulgaricus* CRL 656. Whey reconstituted at a concentration of 16% favored higher growth and enzyme activity, which favors the selection of high-performance strains for the formulation of functional beverages [32].

*α-lactalbumin*, which represents approximately 20% of whey proteins, is a high-quality protein (~14.2 kDa) rich in essential amino acids, especially tryptophan. It plays a key role in lactose biosynthesis through its ability to bind galactose and thereby trigger a conformational change that activates the catalytic complex. Its biological activity is closely linked to its specific folding pattern and the intermolecular interactions that determine both its structural integrity and its functional properties [33,34].

In microbial growth media, the breakdown of α-lactalbumin produces bioactive peptides and free amino acids that serve as nutrients for microorganisms. Research shows that LABs have proteolytic systems that can hydrolyze whey proteins, including α-lactalbumin, to support their growth [35]. Cow’s milk allergens such as α-lactalbumin and β-lactoglobulin can be degraded during fermentation by the proteolytic action of LAB. It has been shown that a combination of *Lactobacillus helveticus* and *Streptococcus thermophilus* significantly reduces the antigenicity of α-lactalbumin within 6 h after fermentation and further decreases it after 12 h of cold storage [36].

α-lactalbumin also exhibits emulsifying properties that are important for microbial growth under lipid-rich conditions. Mutant forms with reduced emulsifying ability have shown impaired microbial proliferation in lipid-containing media, highlighting its role in stabilizing lipid droplets and facilitating nutrient uptake. Furthermore, α-lactalbumin contributes to lipid bilayer integrity, encapsulation efficiency, and the prevention of pore formation, confirming its multifunctionality in both structural and metabolic processes [37].

*Immunoglobulins*, which make up about 5–8% of whey proteins, are glycoproteins that act as antibodies and support the humoral immune response. Cow’s milk contains three primary classes: IgA (300–400 kDa), IgM (around 1030 kDa) [38], and IgG (around 160 kDa [39], with IgG representing about 80% of the total immunoglobulin content. In recent years, there has been increasing interest in the use of bovine immunoglobulins to promote health, particularly in the development of functional foods. Dairy products provide a stable and cost-effective medium for the delivery of these bioactive compounds [40].

The studies showed that a bovine whey-derived immunoglobulin G-enriched powder (IGEP), consisting of 77% protein with 50% IgG content, inhibited the adhesion of *Bifidobacterium longum* subsp. *infantis* to HT-29 intestinal cells at a concentration of 5 mg/mL, while competitive exclusion tests showed a 48% reduction in the adhesion of *Campylobacter jejuni* when IGEP-treated *Bifidobacterium longum* subsp. *infantis* was used [41].

*Glycomacropeptide*, which makes up about 10–15% of whey proteins, is a glycosylated bioactive peptide fragment of κ-casein (between 7.5 and 33 kDa) that is formed during cheese production [42]. It is richer in the essential amino acids threonine, tryptophan, and methionine compared with other whey proteins [43,44,45]. While many studies focused on the inhibitory effect of glycomacropeptides from κ-casein cleavage residues on pathogenic bacterial strains, some studies described glycopeptides that promote the adhesion of bacteria to the intestinal mucosa [44,45,46]. Glycomacropeptides exhibit prebiotic properties by promoting the growth of beneficial intestinal bacteria, especially *Bifidobacterium* species [47].

In human fecal culture systems, glycomacropeptide supplementation stabilized *Bifidobacterium* populations and reduced *Salmonella* counts from log 10^8^ to log 10^7.2^ after 6 days [48]. Animal model studies using male BALB/c mice showed that daily administration of GMP (0.5 mg/mL for 15 days) significantly increased the populations of *Lactobacillus* and *Bifidobacteria* to 2.4 × 10^9^ and 1 × 10^10^ CFU/g, respectively (*p* < 0.01), while *Enterobacteriaceae* and coliforms decreased to 4.5 × 10^7^ and 6.9 × 10^7^ CFU/g, respectively (*p* < 0.05). These results were confirmed by fluorescence in situ hybridization (FISH), which showed that *Lactobacillus* and *Bifidobacteria* accounted for 9.27% and 6.84% of the total gut microbiota in the GMP-treated group, respectively [49]. In allergen-sensitized rats, GMP supplementation increased the number of *Lactobacillus* from 1.21 × 10^9^ to 4.60 × 10^9^ CFU/100 mg feces and the number of *Bifidobacteria* from 4.63 × 10^6^ to 1.50 × 10^7^ CFU/100 mg feces after 10 days of treatment [50]. Glycomacropeptides also support microbial cross-feeding by providing glycans that can be metabolized by *Bifidobacteria*. In GMP-containing culture media, *Bifidobacterium bifidum* JCM1254 showed a significantly higher cell density (OD_600_ = 1.35) compared with glucose medium (OD_600_ = 0.67). Sialidase activity reached 63.64 U/L in GMP media compared with 1.85 U/L in glucose media and facilitated the release of N-acetylneuraminic acid, which decreased by 48% during the growth of *Bifidobacterium breve* [51].

*Serum albumin*, which makes up about 6% of whey proteins, is a globular protein (67 kDa) [52] with high ligand-binding capacity acting as a transporter for fatty acids, hormones, metal ions, and drugs. Studies on bovine serum albumin revealed its unique phase behavior, including reentrant condensation and liquid–liquid phase separation in the presence of trivalent salts such as CeCl_3_. In contrast with human serum albumin, bovine serum albumin did not crystallize under similar conditions due to its weaker hydrophobic interactions. Small-angle X-ray scattering showed lower intermolecular attraction, while adsorption experiments emphasized its ability to form thicker layers on hydrophilic surfaces due to higher hydrophilicity and cation-mediated interactions. Its ability to bind ligands and transport nutrients makes serum albumin a valuable component of microbial culture media, promoting the growth and metabolic activity of various microorganisms [53]. Serum albumin improves the removal of toxins in biotechnological applications by interacting with bacterial cells and modifying surface properties. A combination of 0.5% serum albumin and *Lactobacillus* strains (10^10^ CFU/mL) improved acrylamide removal rates to 35.94% for *Lactobacillus plantarum* 1611 and 30.89% for *Lactobacillus pentosus* ML32. Serum albumin increased surface hydrophobicity, reaching 47.01% and 41.61%, respectively. Simulated gastrointestinal tests showed higher removal rates, up to 41.23%, in intestinal conditions with serum albumin supplementation [54].

*Lactoferrin*, which makes up about around 1% of whey proteins, is a monomeric iron-binding glycoprotein (approximately 78 kDa) composed of a single polypeptide chain. It plays an important role in gut health by modulating the microbiota and promoting beneficial bacteria such as *Lactobacillus acidophilus*, *Bifidobacterium bifidum*, and *Lactobacillus plantarum* [55,56]. Higher fecal lactoferrin levels in neonates have been associated with an increased population of beneficial microorganisms shortly after birth. In preterm infants, lactoferrin supplementation has been shown to reduce the incidence of late-onset sepsis by promoting microbial balance and enhancing the immune response [54]. Mechanistically, lactoferrin sequesters iron and creates a microenvironment that inhibits siderophore-dependent pathogenic bacteria such as *Salmonella typhimurium* and *Escherichia coli*, while favoring iron-independent commensals [56]. In addition to its antimicrobial properties, lactoferrin acts as a prebiotic that selectively promotes the growth of probiotics and improves the integrity of the intestinal barrier through its antibacterial peptides, such as lactoferricin [57]. The concentrations of lactoferrin in human milk—5–7 g/L in colostrum and 2–3 g/L in mature milk—contribute to the maintenance of gut health in newborns [58]. New research based on human and animal models continues to elucidate the mechanisms by which lactoferrin influences the gut microbiota and host physiology [57].

A study using whey- and buttermilk-based formulas enriched with lactoferrin (175 mg/mL) demonstrated its potential to restore the gut microbiota in mice with clindamycin-induced dysbiosis. Supplementation with lactoferrin increased the populations of *Rikenellaceae* (~3000 units) and *Lactobacillaceae* (~6000 units) and restored them to control levels. Functional metabolic pathways such as short-chain fatty acid (SCFA) production and tetrahydrofolate biosynthesis improved significantly, with 21–67 disrupted metabolic pathways restored compared with 10^9^ disruptions in antibiotic-only groups [59].

*Lactoperoxidase*, which represents 0.25–0.5% of whey proteins, is a glycoprotein (78 kDa) known to exert antimicrobial effects against pathogenic microorganisms through catalytic enzyme-mediated reactions that damage pathogenic cells [60].

It catalyzes the oxidation of thiocyanate in the presence of hydrogen peroxide, producing hypothiocyanite, a reactive species that selectively damages pathogenic bacteria while preserving beneficial commensal microbes. This selectivity supports neonatal health and preserves the integrity of the gastrointestinal microbiota. Cow’s milk contains a lactoperoxidase concentration of 2300–5478 mU/mL, which is significantly higher than that of human milk and enables a strong bactericidal effect against pathogens such as *Escherichia coli* and *Staphylococcus aureus*. Synergistically enhanced by xanthine oxidase, which provides hydrogen peroxide, the lactoperoxidase system selectively spares commensal bacteria, supporting neonatal gut colonization and the health of the microbiome [61].

Whey proteins enhance microbial vitality by increasing metabolic activity and serving as substrates for important cell functions. They also facilitate nutrient assimilation, further supporting microbial metabolism. Moreover, their enzymatic hydrolysis produces bioactive peptides that play a key role in stimulating the growth and activity of functional microorganisms [58,62].

### 2.2. Lactose Content

Lactose, the main carbohydrate in whey (4.5–5% of the total composition) is a readily available energy source for microorganisms, especially LABs, and plays a central role in microbial metabolism by influencing growth kinetics, biomass production, and metabolite synthesis [63]. This disaccharide, which consists of glucose and galactose linked by a β-1,4-glycosidic bond, is enzymatically hydrolyzed into its monosaccharide units by β-galactosidase. Glucose enters directly into the glycolytic pathway, while galactose is processed via the Leloir pathway. Together they form an efficient energy source that supports microbial reproduction [64].

The efficiency of lactose metabolism depends on the microbial transport mechanisms. *Lactobacillus* species predominantly use the lactose–phosphoenolpyruvate–phosphotransferase system (PTS), while *Streptococcus thermophilus* uses a permease system, which leads to different metabolic outcomes. *Lactobacillus casei* shows optimal growth at 4–5% lactose and a pH of 6.5, while concentrations below 0.5% become limiting and affect biomass yield and acidification rates [65,66].

Lactose metabolism is also influenced by microbial interactions. *S. thermophilus* preferentially consumes glucose, leaving galactose available for *Lactobacillus bulgaricus*, which promotes symbiotic growth in lactic fermentations [56]. This mechanism of metabolic cross-feeding not only optimizes lactose utilization but also promotes the stability of microbial consortia in whey-based cultures.

Environmental factors additionally modulate lactose metabolism. LABs reach their highest lactose utilization (~95%) at temperatures between 37 and 42 °C and pH values of 5.5–6.5, conditions that optimize glycolytic flux and adenosine triphosphate (ATP) production [67]. The availability of oxygen also plays a role. Although LABs are facultative anaerobes, microaerophilic conditions improve redox, leading to increased lactose metabolism and lactic acid yield [68].

In addition to its role as an energy source, lactose contributes to microbial metabolic diversity by influencing the synthesis of bioactive compounds, organic acids, and exopolysaccharides (EPSs), which improve the functional and probiotic properties of whey-based cultures. These metabolites have potential applications in the food industry, agriculture, and biotechnology.

### 2.3. Minerals and Vitamins

Whey serves as an excellent growth medium for functional microorganisms due to its balanced composition of minerals and vitamins, which provide essential enzymatic cofactors and growth factors. The mineral profile of whey, including calcium (0.4–0.6 g/L), phosphorus (0.4–0.7 g/L), potassium (1.4–1.6 g/L), zinc (1–2 mg/L), iron (0.5–1.0 mg/L), and magnesium (90–120 mg/L), plays a fundamental role in microbial enzyme activity and membrane stability [69].

Calcium supports cell wall integrity and modulates enzymatic activity, with concentrations of 0.5–0.8 g/L increasing *Lactobacillus rhamnosus* and *Bifidobacterium longum* growth rates by up to 25%, likely through the stabilization of β-galactosidase and proteolytic enzymes crucial for lactose metabolism [70]. Additionally, calcium ions promote biofilm formation and enhance probiotic resilience under stress.

Phosphorus, particularly inorganic phosphate, is essential for ATP synthesis and nucleic acid metabolism, with optimal microbial proliferation requiring 0.5–0.8 g/L in high-density fermentations [71]. The calcium–phosphorus ratio modulates nutrient bioavailability, affecting cellular uptake and metabolic efficiency.

Potassium plays an important role in microbial osmoregulation, intracellular pH homeostasis, and the activation of enzymes. As an important intracellular cation, potassium contributes to the maintenance of electrochemical gradients that are important for nutrient transport and metabolic regulation. It facilitates the absorption of important carbon sources, including lactose, by energizing symport mechanisms in the LAB. In addition, potassium-dependent ATPases regulate cellular turgor pressure and ensure structural integrity under osmotic stress conditions [72].

Zinc is an essential trace element that acts as a structural and catalytic cofactor in microbial enzymes including DNA polymerases, proteases, transcription factors, and superoxide dismutase [73]. It regulates gene expression, promotes metabolism, and improves probiotic efficacy in *Lactobacillus plantarum* [74]. Zinc promotes exopolysaccharide production, biofilm formation, and stress resistance of *Lactobacillus* and *Bifidobacterium* [75]. It also supports antioxidant defense and alleviates oxidative stress in *Streptococcus thermophilus* [76].

Iron is an essential micronutrient required for microbial respiration, electron transport chain function, and enzyme activity. Iron is mainly used in its ferrous (Fe^2+^) and ferric (Fe^3+^) forms to facilitate redox reactions in metabolic pathways such as glycolysis and oxidative phosphorylation. Functional microorganism species rely on iron for hem-dependent catalases and cytochromes, which are important for cellular energy production. However, the bioavailability of iron is often limited due to chelation by lactoferrin or interactions with phosphate, so microbial siderophore production is required to improve uptake [77].

Magnesium serves as an important enzymatic cofactor and structural component of ribosomes and plays a crucial role in microbial metabolism and cell stability. It activates numerous enzymes involved in ATP hydrolysis, carbohydrate metabolism, and nucleic acid synthesis. Magnesium also stabilizes cell membranes and ribosomal complexes, thus ensuring efficient protein translation and DNA replication. It also counteracts the inhibitory effects of excess calcium by modulating ion transport systems and reducing competitive interactions at enzymatic binding sites [78].

A well-balanced mineral profile enhances growth kinetics, fermentation efficiency, and functional properties of microbial cultures [79].

Whey also provides water-soluble B-complex vitamins important for microbial metabolism, including riboflavin (B2, 1.5–2.0 mg/L), thiamine (B1, 0.4–0.6 mg/L), and cobalamin (B12, 1.5–2.5 μg/L) [80].

Riboflavin plays an important role in supporting the growth of functional microorganisms cultivated on whey by serving as an important cofactor in redox reactions and energy metabolism. As a precursor of flavin mononucleotide and flavin adenine dinucleotide, it is essential for enzymatic processes in carbohydrate metabolism and ATP production. Riboflavin contributes to protection against oxidative stress by supporting antioxidant defense enzymes, thus maintaining microbial viability. Although riboflavin does not directly drive the biosynthesis of organic acids or bacteriocins, its role in cellular metabolism can indirectly influence the production of these metabolites and thus improve microbial functionality in fermentation processes [81].

Thiamine plays a role in supporting the growth of functional microorganisms cultured on whey by acting as a coenzyme in important metabolic pathways. It is essential for enzymes involved in the pentose–phosphate pathway and the tricarboxylic acid cycle, facilitating carbohydrate metabolism and ATP production [82]. Thiamine also contributes to microbial viability by supporting the cellular functions necessary for growth and adaptation [83].

Cobalamin is an essential cofactor for certain functional microorganisms cultivated on whey, and it supports important metabolic processes. It is required for methionine synthesis by methionine synthase and ensures proper protein production and DNA stability. In bacteria that utilize the methylmalonyl-CoA pathway, cobalamin converts propionate to succinyl-CoA, an important intermediate in central metabolism. Species such as *Lactobacillus reuteri* and *Propionibacterium freudenreichii* rely on cobalamin for optimal growth and metabolic function, which is why its presence in whey-based media is important for fermentation efficiency [84,85].

The synergistic effect of minerals and vitamins in microbial metabolism is well documented, with zinc (1.5–2.0 mg/L) and vitamin B12 (2.0–2.5 μg/L) significantly enhancing exopolysaccharide production in LABs, thereby improving biofilm formation and stress resistance [86]. Likewise, calcium combined with riboflavin reinforces cell membrane integrity and enzymatic stability, increasing probiotic survival under fermentation stress.

The bioavailability of minerals and vitamins directly influences microbial growth efficiency, especially in high-density fermentations. Despite their presence in whey, calcium and magnesium may become limiting factors due to interactions with chelating agents or competing ions, reducing their accessibility to cells [87,88]. Moreover, processing conditions such as heat treatment can degrade vitamins, compromising microbial viability and metabolic performance.

Trace elements also play a critical role in secondary metabolite synthesis, with manganese (0.02–0.05 mg/L) and copper (0.1–0.3 mg/L) modulating antimicrobial compound production in LABs, thereby influencing their probiotic and biopreservative potential [89,90]. Manganese, as a cofactor for superoxide dismutase, enhances oxidative stress tolerance, while copper participates in enzymatic redox reactions that regulate metabolite biosynthesis [91]. Understanding these mineral-dependent metabolic pathways is fundamental to optimizing fermentation efficiency and developing functional dairy products.

By precisely adjusting the micronutrient composition of whey-based media, industrial fermentation strategies can improve microbial viability, probiotic efficacy, and the production of bioactive compounds [92].

## 3. Functional Microorganisms Cultivated in Whey

**Figure 2:**
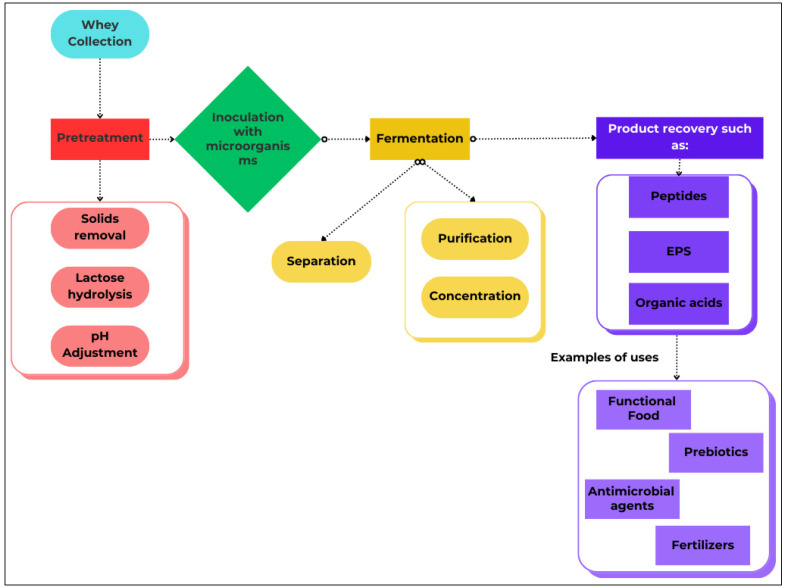
Biotechnological valorization of whey as a fermentation substrate for functional microorganisms. Studies have shown that the most important categories of functional microorganisms that grow in whey are LABs, probiotic bacteria, yeasts, bioremediation bacteria, nitrogen-fixing microorganisms, filamentous fungi, and actinobacteria [10,28,94,95,96,97,98,99] (Table 1).

Whey is an excellent substrate for the cultivation of various microorganisms due to its high content of essential nutrients, including proteins, minerals, and vitamins. To maximize its potential, it is crucial to develop innovative applications for whey that not only prevent spoilage but also promote the growth of desirable microorganisms (Figure 2) [93].

## 4. Techniques for Whey Fermentation, Metabolite Production, and Their Industrial Applications

Functional microorganisms can efficiently convert whey and its components into high-value metabolites using fermentation techniques designed for optimal yield, metabolic efficiency, and scalability. The success of this bioconversion depends on the precise regulation of fermentation parameters such as temperature, pH, inoculum concentration, aeration, agitation, and fermentation time, as these factors directly influence microbial growth, substrate utilization, and metabolite synthesis. Advances in bioprocess control, including automated bioreactor monitoring, pH regulation, oxygen transfer optimization, and real-time metabolic profiling, have significantly improved substrate conversion efficiency, selective metabolite production, and process reproducibility. These support the development of cost-effective, scalable, and industrially sustainable fermentation systems [16,99,135].

**Figure 3:**
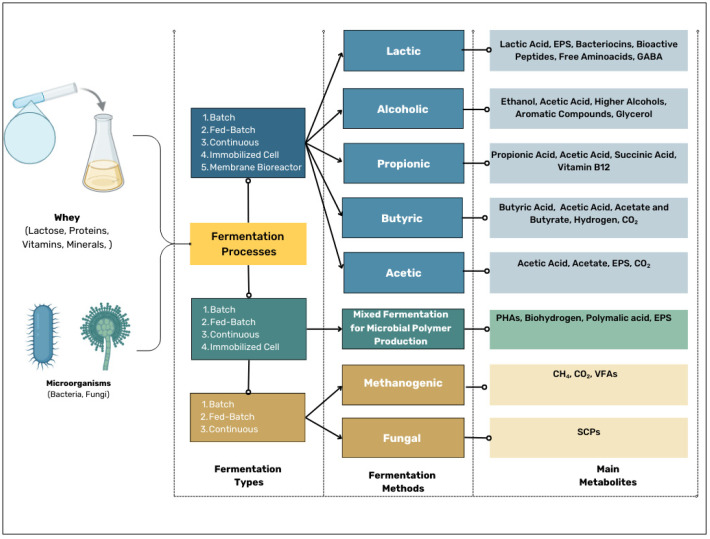
Whey fermentation types, methods, and metabolic outputs.

Whey fermentation methods can be categorized according to their mode of operation, which determines substrate supply, microbial kinetics, process stability, and downstream processing efficiency. Depending on the microbial strain used and the type of fermentation, whey can be converted into a wide range of valuable metabolites, including organic acids, alcohols, biofuels, antimicrobial compounds, biopolymers, and proteins. Each type of fermentation offers different benefits, from fast production times and high product yields to improved metabolic efficiency and minimized by-product formation. Recent studies also show that various whey-derived metabolites—such as organic acids, biopolymers, and bioactive peptides—can serve as building blocks for the development of functional materials and nanomaterials, including smart packaging, wound-healing hydrogels, encapsulation systems, and drug-delivery platforms that have broad applications in the food, pharmaceutical, and biomedical sectors. The following table provides a structured overview of the various methods of whey fermentation and highlights the types of main metabolites produced and their respective industrial applications (Figure 3 and Table 2).

Functional microbial strains cultivated in whey-based substrates have different physiological and metabolic properties that influence their suitability for certain biotechnological applications. *Lactobacillus helveticus* and *Lacticaseibacillus rhamnosus* are known for their pronounced proteolytic activity and their ability to release bioactive peptides with antioxidant and immunomodulatory potential. *Streptococcus thermophilus* shows efficient acidification kinetics and exopolysaccharide (EPS) production, although its tolerance to oxidative stress is relatively limited. *Lactiplantibacillus plantarum* is characterized by robust biofilm formation, stress resistance, and the ability to synthesize EPS with hypocholesterolemic properties. In contrast, *Bifidobacterium animalis* subsp. *lactis* exhibits high colonization potential and health-promoting effects, but its stringent anaerobic requirements may limit its performance under suboptimal oxygen conditions.

Among the non-lactic microbial strains, *Kluyveromyces marxianus* is characterized by its thermotolerance, high lactose assimilation efficiency, and ability to produce ethanol and volatile aromatic compounds, making it valuable for both food and bioethanol fermentation. *Propionibacterium freudenreichii* has unique metabolic capabilities, such as the biosynthesis of vitamin B12 and propionic acid, and it plays a role in modulating the gut microbiota and improving the nutritional quality of fermented products.

The existence of different fermentation types such as batch, fed-batch, continuous, immobilized cell, and membrane bioreactor systems demonstrates the need for tailored bioprocess strategies to optimize microbial growth kinetics and metabolite biosynthesis. Fed-batch systems provide controlled substrate availability to prevent catabolite suppression, while continuous fermentation increases productivity by keeping microbial cultures in an optimal growth phase. Immobilized cell and membrane bioreactor technologies enable prolonged microbial activity, increase volumetric productivity, and reduce downstream processing costs by minimizing cell washout and improving residence time distribution in the bioreactor.

The range of metabolites such as organic acids, alcohols, microbial biopolymers, and gaseous biofuels illustrates the versatility of microbial metabolic pathways in the bioconversion of whey. The industrial applications of whey-derived metabolites are very diverse and include areas such as biotechnology, pharmaceuticals, food and beverages, polymers, biofuels, and sustainable agriculture. This biochemical diversity underlines the importance of whey as a sustainable substrate for the industrial production of high-quality organic products, many of which have already reached market maturity or are in active development. Numerous industrial applications of bioproducts derived from whey have already come onto the market or are under active development. For example, the bacteriocin nisin, which is obtained by the fermentation of whey by *Lactococcus lactis*, is used worldwide as a natural preservative in dairy and meat products and is marketed under the name Nisaplin^®^ (Danisco Limited, 6 North St, Beaminster, UK/DuPont Nutrition Biosciences ApS, Denmark). Bioactive peptides from enzymatically hydrolyzed whey proteins are also used in functional foods and food supplements with antihypertensive, antioxidant, or immunomodulatory properties (Lacprodan^®^ Hydro.365, by Arla Foods Ingredients Group, Viby, Denmark). The lactic acid obtained from whey fermentation is an important starting material in the bioplastics industry, particularly for the production of polylactic acid (PLA), which is used in packaging. Also, EPSs produced by *Lactobacillus* and *Weissella* strains on whey media are being investigated for wound-healing formulations and biodegradable films. In the pharmaceutical and cosmetic fields, lactoferrin extracted from sweet whey is used in antimicrobial creams and iron-enriched formulations (e.g., Apo-Lactoferrin^®^ Athens Research & Technology, Inc. 110, Trans Tech Drive Athens, Georgia, USA). The fermentation of whey with *Kluyveromyces marxianus* or *Debaryomyces hansenii* enables the production of SCPs as sustainable ingredients for animal feed and, more recently, as a meat protein substitute in plant-based foods. The probiotic product Bioenterom, developed using whey as the fermentation substrate and *Enterococcus faecium* NCIMB 11181 as the functional microorganism, is commercially available and recommended in the early life stages of animals to promote beneficial intestinal colonization and inhibit pathogenic bacteria such as *E. coli*, *Salmonella*, and *Clostridium*, being used in poultry, pets, and livestock.

## 5. Limitations and Environmental–Social Impact of Using Whey as a Substrate for Functional Microorganism Cultivation

Whey exhibits considerable variability in composition, which is determined by factors such as the origin of the milk, technological processing, and seasonality. This heterogeneity influences microbial metabolic activity and affects the reproducibility and standardization of fermentation protocols, especially when scaling up from laboratory to industrial conditions. The high content of organic matter and minerals requires precise regulation of key physicochemical parameters, including pH, redox balance, and oxygen availability. Maintaining optimal conditions for microbial growth and metabolite production increases the complexity of the process and requires improved control strategies, which can drive up operating costs. The metabolic performance of functional microorganisms is often strain-specific and requires individual optimization of fermentation conditions to achieve consistent results.

The separation and purification of metabolites derived from fermentation—such as bioactive peptides, exopolysaccharides, and organic acids—pose further technological challenges. Membrane technologies, chromatographic processes, and extraction methods are still limited in terms of scalability, cost efficiency, and environmental compatibility.

Regulatory aspects related to the use of genetically modified organisms or non-traditional microbial strains are insufficiently addressed in the current research. The lack of harmonized safety assessment protocols and standardized methods to evaluate the functionality and safety of novel microbial metabolites limits their wider application in the food and healthcare industries.

Despite these limitations, the use of whey through microbial fermentation brings important environmental and social benefits that reinforce its role as a sustainable resource.

From an environmental perspective, redirecting whey from traditional waste streams into fermentation-based bioprocesses significantly reduces the environmental footprint of dairy by-products. Untreated whey poses a serious threat due to its high BOD and COD, contributing to oxygen depletion and eutrophication of water bodies. Fermentation mitigates these risks while reducing the energy requirements and emissions associated with conventional treatment processes. Life cycle assessment (LCA) studies report a reduction in environmental impact of up to 65% when whey is converted into biofuels, biopolymers, or other high-value products instead of being disposed of directly. Integrating strategies such as recycling process water and optimizing land use can improve resource efficiency and support biodiversity conservation.

On a social level, the utilization of whey supports regional economic development by promoting knowledge-based jobs in rural and semi-rural areas that produce milk. Bioprocessing facilities demand skilled labor, offering employment opportunities beyond those of the traditional waste industry. This has a positive socio-economic impact on local communities. The conversion of whey into functional foods or SCP components offers accessible and sustainable protein alternatives with less environmental intensity than traditional animal proteins. This contributes directly to global food security. At the same time, reducing the runoff of untreated whey improves environmental hygiene and reduces the incidence of waterborne diseases, thereby improving public health in vulnerable areas near dairy farms.

## 6. Challenges and Future Perspectives

The use of whey as a substrate for the cultivation of functional microorganisms and the production of bioactive metabolites poses challenges in terms of process efficiency, strain optimization, and industrial scalability. The variability of whey composition due to processing methods and seasonality affects microbial metabolism and requires strict standardization to ensure consistent bioconversion yields. The high organic load of whey requires precise control of pH, oxygen transfer, and nutrient availability to maintain process stability and optimize microbial kinetics.

**Figure 4:**
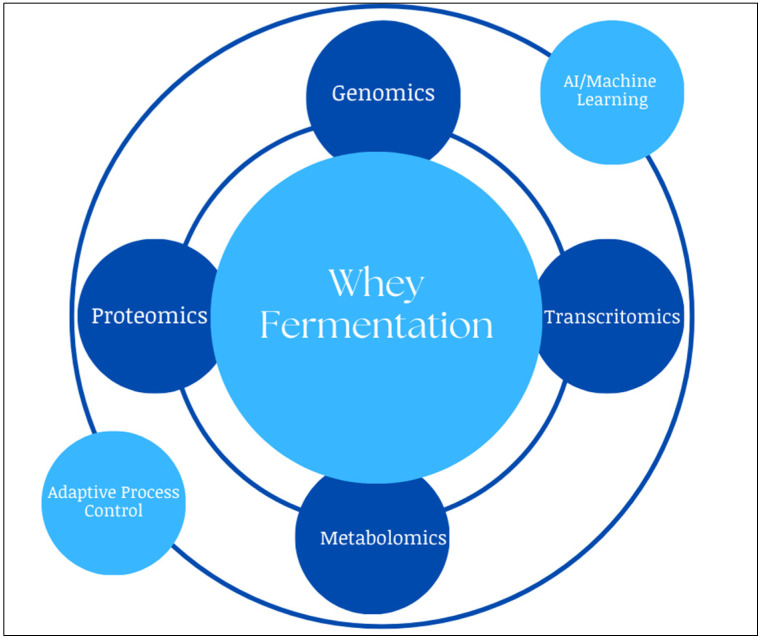
Integrating omics and AI in whey fermentation process optimization.

Economic feasibility is often hindered by costly downstream processing, as the purification of compounds such as bacteriocins, EPS, and organic acids requires advanced filtration, chromatography, or extraction methods. Improving efficiency through membrane-integrated bioreactors, in situ product recovery, and solvent-free purification is essential for industrial-scale applications. To address these challenges, recent advances in multi-omics technologies and computational modeling have enabled more precise control of fermentation processes and microbial metabolism (Figure 4).

These tools allow the detailed characterization of microbial dynamics and the regulation of metabolic pathways and stress responses during whey fermentation. The integration of omics data with machine learning improves predictive modeling and enables real-time adaptive control that accounts for substrate composition variability. Engineered microbial consortia with complementary metabolic functions further improve co-fermentation and substrate conversion.

The future of whey-based microbial bioprocessing lies in the convergence of metabolic engineering, systems biology, and bioprocess intensification strategies. The identification and optimization of novel bioactive metabolites such as functional peptides, antimicrobial proteins, and secondary metabolites with pharmaceutical potential will further increase the industrial importance of whey bioconversion. By using synthetic biology tools and precision fermentation techniques, microbial metabolism can be fine-tuned to improve yield and specificity. By integrating state-of-the-art biotechnological advances with sustainable process design, whey fermentation can be established as the cornerstone of next-generation microbial bioproduction and drive innovation in bio-based materials, food supplements, and biopolymers while strengthening environmental sustainability.

Despite these promising directions, there are still some research gaps and methodological limitations in the literature that are not sufficiently addressed. Most available studies are limited to monocultures of well-characterized lactic acid bacteria or model yeast strains under controlled laboratory conditions. There is a lack of comparative, large-scale data to evaluate co-culture dynamics, strain compatibility, and long-term process stability in whey-based systems. In addition, standardization is hindered by the lack of uniform protocols for evaluating metabolite yield, bioactivity, and microbial viability, making comparisons between different studies difficult. There is also controversy regarding the regulatory acceptance of bioactives produced by engineered strains or unconventional microorganisms. Moreover, there are few techno-economic and environmental analyses, although they are important for industrial feasibility and the integration of the bioeconomy into the circular economy. To overcome these limitations, a systematic and interdisciplinary research approach combining high-throughput screening, omics-based metabolic mapping, process modeling, and life cycle assessment is needed to identify critical bottlenecks and enable scalable, safe, and economically viable fermentation platforms.

## 7. Conclusions

Whey valorization is increasingly recognized not only as a cost-effective substrate but also as a strategic enabler for sustainable biotechnologies. Beyond its traditional applications, whey supports advanced microbial production systems that are in line with the goals of the circular bioeconomy.

New research directions focus on precision fermentation using multi-omics and computational tools that open up avenues for the targeted synthesis of high-value compounds. Equally promising is the use of underutilized whey fractions and the rational assembly of synthetic microbial consortia with the aim of diversifying substrate use and improving process outcomes.

These innovations enhance the potential of whey to contribute to rural development, sustainable food systems, and low-carbon industries. As the field advances, more attention is needed to create regulatory frameworks and policy support mechanisms that facilitate the introduction of microbial-derived ingredients at scale.

Overall, whey is proving to be a renewable and versatile feedstock for next-generation bioprocessing that is well positioned to meet future sustainability and bioeconomy goals.

## Figures and Tables

**Table 1:** Functional microbial categories that utilize whey as a growth substrate.

Categories of Functional Microorganisms/Genus	Species Cultivated on Whey as Medium	Main Metabolic Activity	Optimal Temperature Range	Advantages	References
**LABs** *Lactobacillus* *Streptococcus* *Lactiplantibacillus* *Limosilactobacillus* *Lacticaseibacillus* *Lactococcus* *Enterococcus* *Leuconostoc*	*Lactobacillus fermentum* *Lactobacillus delbrueckii* *Lactobacillus helveticus* *Streptococcus thermophilus* *Lactococcus lactis*	Lactose fermentation, proteolysis, production of EPS and other bioactive metabolites	37–42 °C	- Tolerant to acidic conditions; found in spontaneous whey cultures - Strong proteolytic activity; synergistic with *S. thermophilus* for bioactive peptide production - High proteolytic activity; produces antihypertensive and antioxidant peptides; isolated from natural whey cultures - Synergistic with *L. delbrueckii* in mixed fermentations - Used in food-grade fermentations; adapts well to whey	[13,35,100,101,102]
**Probiotic bacteria** *Lactobacillus, Lactiplantibacillus, Limosilactobacillus, Lacticaseibacillus, Lactococcus, Enterococcus* *Bifidobacterium* *Propionibacterium* *Bacillus* *Pediococcus* *Weissella*	*Lactobacillus acidophilus* *Lactobacillus fermentum* *Lactobacillus helveticus* *Lactobacillus delbrueckii subsp. bulgaricus* *Lactiplantibacillus plantarum* *Limosilactobacillus reuteri* *Lacticaseibacillus casei* *Lacticaseibacillus rhamnosus* *Lactococcus lactis* *Enterococcus faecium* *Bifidobacterium animalis subsp. lactis* *Propionibacterium freudenreichii* *Bacillus subtilis* *Pediococcus acidilactici*	Lactose fermentation, proteolysis, pH regulation and acidification of the gut environment, production of bioactive metabolites Fermentation of lactose and oligosaccharides, production of SCFAs, vitamin production Fermentation of lactose and lactate, production of propionic acid, production of vitamin B12 Production of extracellular enzymes, proteolysis Fermentation of carbohydrates, production of bacteriocins	37–42 °C 37–45 °C around 30 °C 30–39 °C 30–37 °C	- Commonly used in mixed fermentations with other LABs to enhance the functional value of dairy products - Tolerant to acid and osmotic stress; enhanced growth and metabolic activity in whey-based optimized media - Strong proteolytic activity; produces antihypertensive and antioxidant peptides; frequently isolated from natural whey cultures - Thermophilic strain with synergistic action with *Streptococcus thermophilus* - Highly versatile in whey fermentation; prebiotic effect, antioxidant, and cholesterol-lowering properties - Demonstrates stability after spray-drying and microencapsulation; suitable for development of shelf-stable probiotic formulations - Highly adaptable to lactose-rich media - Strong lactose fermentation capacity; known for biofilm formation and high survival in functional foods - Widely used in food-grade fermentations; well adapted to different whey types - Potential probiotic activity - Widely used in probiotic dairy products - Suitable for functional food development due to antimicrobial and metabolic benefits - Ferments efficiently in dairy by-products; survives well in freeze-dried probiotic formulations - Used as natural food preservation	[13,103,104,105,106,107,108]
**Bioremediation bacteria** *Bacillus* *Streptomyces* *Dehalococcoides*	*Bacillus methylotrophicus* *Streptomyces* sp. *Dehalococcoides* sp.	Production of biosurfactants, degradation of organic compounds, production of enzymes, production of antimicrobial compounds, degradation of organochlorinated compounds	30 °C 25–30 °C	- Suitable for eco-friendly soil bioremediation - Grown on whey to improve compost quality and microbial biomass; contributes to organic matter stabilization and nutrient cycling during composting - Activity enhanced in whey-fed bioremediation systems, particularly in sulfate-rich aquifers	[104,109,110,111]
**Nitrogen-fixing microorganisms** *Rhizobium*	*Rhizobium meliloti*	Nitrogen fixation, fermentation of carbohydrates	30 °C	- Capable of large-scale biomass production using whey as a cost-effective substrate; suitable for bioinoculant formulations in agriculture	
**Actinobacteria** *Streptomyces* *Corynebacterium* *Rhodococcus* *Arthrobacter* *Brevibacterium*	*Streptomyces* sp. *Corynebacterium glutamicum* *Rhodococcus opacus* *Arthrobacter agilis* *Arthrobacter viscosus* *Brevibacterium linens*	Production of biosurfactants, amino acids (lysine) production, lactose fermentation into ethanol, production of lipids Production of C50 carotenoids of the bacterioruberin type and its glycosylated derivatives, production of EPS, Metabolism of lactic acid and proteins, production of ammonia and carotenoids, inhibition of pathogenic microorganisms	30 °C 30–37 °C 28–30 °C 20–30 °C 25–30 °C	- Enhances compost microbial structure and nutrient availability; supports organic waste stabilization - Engineered strains use lactose and galactose from whey to produce L-lysine - Exhibits high lipid accumulation rates - Sustainable for pigment production - Improves rind texture and suppresses *Listeria* and other spoilage microorganisms during cheese maturation - Improves flavor development and microbial stability in smear-maturation cheeses	[99,112,113,114,115,116,117]
**Yeasts** *Kluyveromyces* *Yarrowia* *Candida* *Debaryomyces* *Rhodotorula* *Saccharomyces* *Torulopsis*	*Kluyveromyces lactis* *Kluyveromyces marxianus* *Candida tropicalis* *Debaryomyces hansenii* *Rhodotorula glutinis*	Fermentation of carbohydrates, production of enzymes, production of aromatic compounds, production of lipids, production of carotenoids	30–45 °C 30–37 °C 25–30 °C 28–30 °C	- Suitable for ethanol and lactic acid production from sweet and acid whey; grows well under mild industrial conditions - Thermotolerant and fast growing; highly scalable for industrial bioprocesses - Enhances proteolysis; useful for flavor enhancement - Suitable for recombinant protein production using salt-rich dairy by-products - Capable of synthesizing carotenoids (e.g., β-carotene, torulene) when grown on goat cheese whey	[118,119,120,121,122,123,124,125]
**Filamentous fungi** *Aspergillus* *Penicillium* *Trichoderma* *Mucor* *Fusarium* *Rhizopus* *Geotrichum*	*Aspergillus oryzae* *Aspergillus niger* *Aspergillus flavus, Aspergillus awamori, Aspergillus tubingensis* *Aspergillus tamarii* *Penicillium chrysogenum* *Penicillium roqueforti* *Penicillium camemberti* *Penicillium brevicompactum* *Trichoderma harizanum* *Mucor genevensis* *Mucor circinelloides* *Mucor azygosporus* *Mucor miehei* *Fusarium semitectum* *Fusarium solani* *Fusarium culmorum* *Fusarium oxysporum kolhapuriensis* *Rhizopus oryzae* *Rhizopus arrhizus* *Geotrichum candidum*	Fermentation of lactose, proteolysis, lipolysis, production of enzymes, production of secondary metabolites, degradation of organic compounds Production of lipids, production of organic acids	25–37 °C 20–28 °C 25–30 °C 30–37 °C 25–30 °C	- Suitable for animal feed and enzymatic applications Recombinant strains used for enzyme production in industrial processes - Used for enzyme production - Used for protease production in dairy media - Used for the production of active metabolites and secondary compounds on agro-industrial residues including whey - Used in cheese ripening and flavor development involved in rind formation and aroma development in soft cheeses - Applicable in food colorants and functional ingredient production - Used as a biocontrol agent; applicable in biofertilizer production - Capable of acid-whey deacidification and lipid accumulation - Involved in biomass valorization for lipase production - Applicable in biofuel and nutritional lipid production - Involved in studies on oil accumulation for industrial uses - Used for Single-Cell Proteins (SCPs) production and deacidification of acid whey - Involved in cheese surface microbiota; contributes to rind development and deacidification	[10,126,127,128,129,130,131,132,133,134]

**Table 2:** Main metabolites produced by whey fermentation and their industrial applications.

Fermentation Methods	Main Metabolites Produced	Functional Properties Highlighted in Various Studies	Industrial Applications	References
Lactic fermentation	Lactic acid	Lactic acid inhibits Propionibacterium acnes at concentrations above 60 mg/mL, which supports its role in the treatment of acne and skin whitening. Its efficacy may vary depending on the stability and concentration of the formulation.	Food preservation, dairy products, bioplastics, cosmetics, pharmaceuticals	[13,38,99,136,137,138,139]
	EPS	EPSs exhibit a wide range of strain-specific bioactivities: EPS from *Leuconostoc pseudomesenteroides* increased *Lactobacillus* counts by 20% and reduced *E. coli* by 15% in rats; EPS from *Bifidobacterium longum* reduced lung eosinophils by 40% in an asthma model; sulfonated EPS from *Lactiplantibacillus plantarum* increased antioxidant activity by 35%, while EPS from *L. lactis* subsp. *cremoris* lowered cholesterol by 25% in rats. HePS inhibited biofilms by 70%, and *Lactobacillus gasseri* EPS suppressed *E. coli*, *L. monocytogenes*, and *S. aureus* by 60%. EPS-loaded nanoparticles reduced tumor volume by 70%, indicating a promising but context-dependent therapeutic potential.	Functional foods, dairy products, prebiotics, biopolymers	[140,141,142,143,144]
	Bacteriocins	Bacteriocins exhibit strong antimicrobial activity, positioning them as effective natural food preservatives. *In situ* production using starter cultures is promising for fermented foods; however, their application still depends on strain compatibility and product matrix. Commercial examples include *Lactococcus lactis* subsp. *lactis* BS-10 (BioSafe™) for cheese and *Leuconostoc carnosum* (Bactoferm™ B-SF-43) or *Lactobacillus sakei* (Bactoferm™ B-2) for vacuum-sealed meat. Lacticin 3147 has been shown to improve the quality of cheddar cheese by controlling non-starter LABs. Bacteriocin-producing LABs are also used to preserve plant-based foods, seafood, and fermented vegetables, although their wider industrial use may be limited by production stability, regulatory approval, and interaction with complex food systems.	Food preservation, pharmaceuticals, antimicrobial coatings	[145,146,147,148,149,150,151,152,153]
	Bioactive peptides and free amino acids	The antihypertensive effect of peptides from whey is primarily associated with angiotensin-converting enzyme (ACE) inhibition, with whey protein hydrolysate lowering systolic blood pressure by 30% in hypertensive rats. Co-fermentation of *Lactobacillus paracasei* and *Saccharomyces cerevisiae* has shown that novel ACE inhibitory and antioxidant peptides can be produced from whey protein concentrate, increasing serum antioxidant capacity by 40%. These peptides also showed antimicrobial activity by inhibiting the growth of *Listeria* and *E. coli* by 80% at 0.2 mg/mL and increasing the proliferation of immune cells by 50% in vitro. While these multifunctional properties suggest considerable therapeutic potential, their efficacy and stability in various biological systems and routes of administration need to be further validated for practical applications.	Functional foods (infant formula, sports nutrition, medical nutrition), nutraceuticals, pharmaceuticals	[145,154,155,156,157,158]
	γ-aminobutyric acid (GABA)	Fermented rice flour that contains 750.55 ± 26.03 mg GABA/100 g has been shown to reduce oxidative stress and improve neuroprotection. GABA also contributes to blood pressure regulation, as shown by a reduction in systolic pressure of 5.5 ± 3.9 mmHg over 12 weeks after daily consumption of 50 g cheddar cheese containing 16 mg GABA. A single dose of chocolate enriched with 28 g GABA from *Lactobacillus hilgardii* K-3 reduced stress levels. These results underline the multifunctional health potential of GABA. The physiological effects of GABA may vary depending on delivery matrix, dosage, and individual response, so further studies on standardized use in functional foods are needed.	Functional beverages, food supplements, pharmaceuticals	[159,160,161,162,163]
Alcoholic fermentation	Ethanol	Fermentation studies have reported ethanol concentrations reaching 4.52% after 20 days, though such yields are strongly influenced by fermentation conditions and substrate composition. *Kluyveromyces marxianus* produces ethyl acetate under aerobic conditions, which can be economically recovered from bioreactors for use in flavors and solvents. Recent studies indicate that metabolite production by *K. marxianus* is significantly dependent on oxygen availability, medium composition, and fermentation mode, which may affect process consistency and scalability in industrial applications.	Bioethanol production, pharmaceutical solvents, beverages	[164,165,166,167,168,169,170]
	Acetic acid	*Dekkera anomala* thrives under aerobic conditions and produces acetic acid with remarkable antimicrobial and preservative activity. Fermentation with *D. anomala* yields 9.18 g/L acetic acid after 34 days, supporting its potential use in food preservation at concentrations of 0.1–0.5%. Ethanol and acetic acid produced by these yeasts serve as effective antimicrobial agents; ethanol from whey-based disinfectants significantly reduced bacterial contamination, while acetic acid inhibited *Listeria monocytogenes* and *E. coli* in food matrices. *L. monocytogenes* was reduced by 90% at 0.5% and 99.9% at 2% acetic acid, while *E. coli* was reduced by 99% at 1.5% and 99.9% at 3%. A 2.5% acetic acid solution reduced *L. monocytogenes* in meat products by 3.7 log CFU/g after 24 h at 4 °C. Despite these promising results, the relatively slow fermentation rate and the concentration-dependent efficacy underline the need for process optimization and product-specific validation in industrial applications.	Food industry, pharmaceuticals, polymers, textiles, agrochemicals, cosmetics, chemical industry	[171,172,173,174,175,176,177,178]
	Higher alcohols and aromatic compounds	Higher alcohols from whey fermentation improve sensory properties and show antimicrobial activity. Isoamyl and phenylethyl alcohol produced during alcoholic fermentation inhibit spoilage organisms—phenylethyl alcohol at a concentration of 0.3% suppresses ~95% of *Zygosaccharomyces bailii*, while isoamyl alcohol at a concentration of 0.5% inhibits 90% of *Penicillium* species. Although these effects are promising, their application may be limited by concentration thresholds and product compatibility.	Biofuels, alcoholic beverages, chemical synthesis; dairy industry, flavoring agents, bakery products	
	Glycerol	Glycerol is frequently used in pharmaceutical formulations and cosmetics due to its moisture-retaining and stabilizing effect in concentrations of 5–30%. In biotechnology, it acts as a metabolic regulator at 0.5–10% (*w*/*v*) and can be converted by microbial fermentation into high-value products such as 1,3-propanediol and polyhydroxyalkanoates (PHAs). Although its multifunctionality is well documented, optimization of conversion efficiency remains essential for broader industrial integration.	Cosmetics, pharmaceutical stabilizers, food processing	
Propionic fermentation	Propionic acid	Propionic acid serves as a natural preservative for food and effectively inhibits mold growth in baked goods and dairy products. Studies indicate strong antifungal activity, particularly against *Aspergillus fumigatus*, *Penicillium roqueforti*, *Pichia anomala*, and *Kluyveromyces marxianus*, with minimum inhibitory concentration (MIC) values between 20 and 120 mM at pH 5 and below 10 mM at pH 3. *P. roqueforti* was the most sensitive, showing inhibition at 50 mM even at pH 7. While the pH-dependent efficacy highlights its potential, optimization of formulation conditions is key to ensuring consistent antifungal performance in different food systems.	Food preservatives, bioplastics, functional foods, nutraceuticals, pharmaceuticals, feed supplements, cosmetics agrochemicals	[12,179,180,181,182,183,184,185,186,187]
	Acetic acid	Acetic acid shows remarkable antimicrobial activity, with MIC values of 30–120 mM at pH 5 and effective suppression of fungal growth. The enhanced antifungal activity observed when combined with other organic acids, such as propionic acid, highlights its potential for natural food preservation, although the synergy of the formulation and pH sensitivity require careful optimization for consistent efficacy.	Food industry, pharmaceuticals, polymers, textiles; agrochemicals, cosmetics, chemical industry	
	Succinic acid	Succinic acid from whey fermentation can achieve yields of 25–30 g/L with over 98% purity after processing. It is used as a flavor enhancer, acidity regulator, and gelling agent in foods, while succinic acid and its derivatives, such as sodium succinate, are used in packaging and as slow-raising agents. As an important precursor for biodegradable plastics, it supports the production of co-polyesters for sustainable food packaging. It also acts as an ion chelator and surfactant in pharmaceuticals and as an acidifier and stabilizer in cosmetics. Despite its versatility, wider industrial application may depend on cost-efficient production and integration into existing manufacturing processes.	Polymers, pharmaceuticals, food industry, cosmetics, chemical industry, agriculture	
	Vitamin B12	*Propionibacterium freudenreichii* subsp. *shermanii* efficiently synthesizes vitamin B12 under optimal conditions: pH 6–7, lactate as carbon source, and fed-batch fermentation. Whey is a cost-effective medium when using a 24 h inoculum, 5 mg/L iron, 4% lactose, and 0.5% (NH_4_)_2_HPO_4_, with fermentation typically involving anaerobic and aerobic phases at 30 °C. While yields are promising, maintaining these specific parameters is critical for consistent B12 production on a large scale.	Pharmaceuticals, nutraceuticals, food industry, animal nutrition, biotechnology, cosmetics	
Butyric fermentation	Butyric acid	Butyric acid has been shown to be effective against pathogens such as *Salmonella* and *E. coli* while supporting beneficial gut microbiota. Supplementation with 0.63 g/kg reduced *Salmonella* colonization in chickens, and 0.4% in broilers improved weight gain and feed conversion, and reduced E. coli levels, comparable to antibiotics. This dose also lowered pH in the upper part of the digestive tract, increased villus length and crypt depth, improved carcass yield, and reduced abdominal fat. While these results highlight its potential as an alternative to antibiotics in poultry feed, consistent performance under variable rearing conditions is worth further validation.	Polymers, chemical industry, food and nutraceutical industry, feed industry, pharmaceuticals, bioenergy	[188,189,190,191]
	Acetic acid	Acetic acid strongly inhibits *Saccharomyces cerevisiae* and outperforms sorbic acid at higher concentrations. Its antifungal effect is pH dependent: 100–115 mM completely inhibited *Fusarium oxysporum* at a pH of 4.8 (tomato), and 45–60 mM reduced spore germination of *Penicillium expansum* by 78–86% at a pH of 4.2 (apple juice). Although the effect is effective in acidic foods, the results may vary depending on the matrix and strain.	Food industry, pharmaceuticals, polymers, textiles, agrochemicals, cosmetics, chemical industry	[192,193,194,195]
	Acetate and butyrate	Clinical studies report that 2–5 g/day of butyrate increases *Faecalibacterium prausnitzii* by 28–47% and reduces inflammatory markers by 23–35%. Acetate at a concentration of 10–30 mM in the colon decreases *E. coli* adhesion by 42%. In bioplastics, acetate-based PHAs (15–25% acetate) are 76% biodegradable within 180 days. In livestock, 0.3–0.5% sodium butyrate improves poultry feed conversion by 8.7–12.3% and reduces intestinal pathogens by 18.5%. Acetate at 1–3 g/kg increases soil microbial biomass by 27% and organic carbon by 9–15%. Both acids are used as industrial solvents, with global production of acetate and butyrate exceeding 3.2 million and 80,000 tons, respectively. Despite their broad functionality, application efficiency varies by sector and depends on formulation, dosage, and environmental conditions.	Food and nutraceutical industry, pharmaceuticals, bioplastics, chemical industry, agriculture, feed industry, biofuels	[193,196,197,198,199]
	Hydrogen	Fermentative hydrogen production yields 1.5–2.8 mol H_2_/mol glucose with 15–24% conversion efficiency in optimized bioreactors. It plays a key role in biofuels and industrial cooling, where hydrogen systems operate at –253 °C with a coefficient of performance of 0.3–0.5. Blending hydrogen with natural gas (5–20%) reduces carbon emissions from heating by 7–13%. In the chemical industry, green hydrogen reduces the carbon footprint of ammonia synthesis by 65–90%, with modern Haber–Bosch plants consuming 28–37 GJ/ton of ammonia. Despite its potential, scalability and energy consumption remain critical factors for sustainable use.	Renewable energy, chemical industry, fertilizers	[200,201,202]
	Carbon dioxide (CO_2_)	CO_2_, a by-product of fermentation, is often used in the food industry to carbonate beverages at 3.5–7.0 g/L. In refrigeration, it is considered an environmentally friendly alternative with a GWP of 1, far lower than conventional refrigerants (GWP 1430–4000), and it achieves energy efficiency values of 2.5–3.8 in transcritical cycles. In greenhouse agriculture, CO_2_ enrichment at 800–1200 ppm increases tomato yields by 27–42% and lettuce biomass by 25–35% compared with ambient levels (~410 ppm). While the benefits are well documented, the efficiency of implementation depends on system design, cost, and sector-specific integration.	Food industry, industrial refrigeration, greenhouse agriculture	[203,204,205,206]
Acetic fermentation	Acetic acid	Acetic acid is widely known for its antimicrobial properties. It reduces *E. coli* O157:H7 by 99.7% at 0.1–0.5% in 24 h and lowers *Listeria monocytogenes* by 3–5 log CFU/g at 2–3% in meat. In pharmaceuticals, 0.25–1.0% solutions inhibit *Pseudomonas aeruginosa* at MICs of 0.16–0.31%. It is also used in acetate fibers (25–30 MPa tensile strength; >1.5 Mt/year production) and cosmetics (0.1–0.5%) to regulate pH (3.5–5.0) and improve the skin barrier by 18–27%. With a global production of over 13.8 Mt/year, acetic acid is an important chemical intermediate. Despite its versatility, its efficacy and safety depend on the exact formulation and context of use.	Food industry, pharmaceuticals, polymers, textiles, agrochemicals, cosmetics, chemical industry	[207,208,209,210,211,212,213]
	Acetate	An acetate content of 60–80 mM in the gut increases butyrate-producing bacteria by 35–40% and reduces inflammatory markers by 25%. In pharma, sodium acetate buffers enhance protein stability by 28–42%. Acetate-based PHAs (15–25%) show 76–90% in soil within 180 days. In animal feed and agriculture, 0.2–0.5% acetate improves digestion, increases feed conversion in poultry by 7–12%, and reduces CH_4_ emissions in ruminants by 15–22%. The chemical industry uses acetate in solvents, adhesives, and coatings, with a global production of over 3.5 Mt/year. Although it is versatile, its effectiveness depends on the formulation and sector-specific conditions.	Food and nutraceutical industry, pharmaceuticals, bioplastics, chemical industry, agriculture, feed industry	[214,215,216]
	EPS	EPS increases the viscosity of yogurt by 150–300% at 0.05–0.2% and reduces syneresis by 45–60%. In pharmaceuticals, 1.5–3 g/day increases SCFA production by 24–36% and bifidobacteria by 18–27%. In biotechnology, 2–5 g/L EPS increases the binding of heavy metals by 65–80% through biofilm formation. Biomedical applications include wound healing and drug delivery, where EPS hydrogels improve wound closure by 40–55% and prolong drug release by 30–45%. Despite their broad functionality, their efficacy is context- and formulation-dependent.	Food industry, pharmaceuticals	[217,218,219,220]
	CO_2_	Controlled CO_2_ levels can enhance the productivity of industrial fermentation by 15–30% by optimizing dissolved CO_2_ and improving kinetics and metabolite synthesis. Specific CO_2_ concentrations also reduce unwanted by-products by 25–40% through effects on metabolic flux and substrate uptake. In penicillin production, precise CO_2_ control increases antibiotic titers by 18–24% and improves microbial performance. In addition, CO_2_ optimization reduces energy consumption by 12–19% through improved glucose–oxygen efficiency. While these results are promising, they depend on tightly controlled process conditions.	Food industry, biotechnology	
Mixed fermentation for microbial polymer production	PHAs	PHAs containing 10–30% hydroxyvalerate exhibit greater flexibility, with elongation at break increasing from 5–10% to 30–450% compared with pure PHB, making them ideal for bioplastics. In tissue engineering, PHA-based scaffolds improve cell adhesion and proliferation. They exhibit 75–90% better biocompatibility and 65–85% higher cell viability than conventional synthetic polymers. PHAs with 10–30% hydroxyvalerate show increased flexibility, with elongation at break rising from 5–10% to 30–450% versus pure PHB, supporting their use in bioplastics. In tissue engineering, PHA scaffolds enhance cell adhesion and proliferation, with 75–90% better biocompatibility and 65–85% higher viability than synthetic polymers. Despite these advantages, performance can vary with composition and application context.	Polymers	[141,221,222,223,224]
	Biohydrogen	Biohydrogen is gaining relevance as a renewable energy source, with fermentative yields of 1.8–2.5 mol H_2_/mol glucose under optimized conditions. Hydrogen-enriched fuels (15–20% H_2_) reduce CO_2_ emissions by 10–15% and NOx by 20–50%. Microbial reactors achieve up to 40% energy efficiency and produce 0.5–3.5 L H_2_/day in continuous operation. Practical scalability depends on whether these performance levels can be maintained in real systems.		[225,226,227,228]
	Polymalic acid (PMA)	PMA, a biodegradable polyester, is widely studied for biomedical and pharmaceutical purposes. PMA nanocarriers enable 50–70% extended drug release over 7–21 days with 15–30% loading efficiency. In bioplastics, PMA films offer a tensile strength of 20–35 MPa and an oxygen permeability of 2.5–8.7 cm^3^-mm/m^2^-day-atm, making them suitable for sustainable packaging. During composting, PMA biodegrades 85–95% within 90 days. Despite its promising properties, performance can vary depending on the formulation and application environment.		
	EPS	EPS-based hydrogels improve water retention by 60–80%, retain 20–35 g water/g dry matter, and are suitable for biomedical dressings and agriculture. In biopolymers, EPS improves the flexibility of films by reducing the modulus of elasticity by 30–45%. Plastics with 5–15% EPS degrade 85% faster and achieve 90–95% biodegradation in 60–180 days compared with more than 365 days for conventional plastics. While the effect is effective, performance can vary depending on matrix compatibility and environmental conditions.		
Methanogenic fermentation	CH_4_	CH_4_ is an important renewable fuel in biogas production, with optimized anaerobic digestion of whey yielding 0.3–0.5 m^3^ CH_4_/kg COD. CH_4_-fueled engines reduce CO_2_ emissions by 15–25% compared with diesel, while bio-CNG in public transportation reduces NOx by 40–60% and particulates by 85–95%. Biogas plants using cheese whey achieve 0.34–0.48 m^3^ CH_4_/kg COD, with 55–68% energy recovery and 3.5–7 years payback. Despite strong potential, the results depend on the plant scale and process optimization.	Renewable energy, fuel for heating, transportation	[16,229,230,231,232,233,234,235,236]
	CO_2_	Optimized CO_2_ levels in sparkling water (5.8–6.2 g/L) increase consumer preference by 32–41%, while sparkling wines require more than 1.2 g/L for perceptible mouthfeel. At retail, transcritical CO_2_ systems reduce energy consumption by 18–26%, with advanced designs (e.g., two-phase ejector, heat exchanger) improving COP by 12% and energy efficiency by 16% and reducing electricity consumption by 10%. Cascade CO_2_ systems achieve an efficiency of 3.2–4.1 for medium-temperature cooling. In biotechnology, controlled CO_2_ increases the expression of recombinant proteins by 25–33%. Although the benefits are significant, system design and application context remain critical for consistent performance.	Food industry, industrial refrigeration, greenhouse agriculture	
	Volatile fatty acids (VFAs)	VFAs can be converted to PHAs with yields of 0.2–0.6 g PHA/g VFA. Industrial processes using mixed VFAs from wastes achieve 50–150 g/L PHA and 1.0–3.5 g/L/h productivity. The resulting PHAs have a tensile strength of 20–40 MPa and an elongation at break of 5–500%, with an oxygen permeability of 3–55 cm^3^-mm/m^2^-day-atm and a water vapor permeability of 1–5 g-mm/m^2^-day, suitable for sustainable packaging. VFAs also support the production of solvents and coatings, with acetate to ethanol conversion at 85–92% and propionate-based adhesives achieving bond strengths of 2.5–4.8 MPa. While performance and efficiency are promising, they are highly dependent on feedstock variability and process control.	Polymers	
Fungal fermentation for SCPs production	SCPs	SCPs-enriched bread formulations with 5–10% fungal protein increase the protein content by 18–25%, with sensory acceptability at 85% of control products. SCP (mycoprotein) derived from *Fusarium venenatum* has been shown to reduce postprandial blood glucose levels by 20–35% and insulin response by 15–27% compared with reference proteins, thus supporting metabolic health. In the animal feed industry, SCPs are used as a high-protein supplement for livestock and aquaculture. Replacing 20–40% of fishmeal with SCPs from *Aspergillus oryzae* in aqua feed improves fish growth rates by 10–18% and increases feed conversion in tilapia and rainbow trout by 12–20. In poultry, the inclusion of 5–15% fungal SCPs in feed increases weight gain by 8–14% while improving gut health by reducing *Salmonella* by 22–30% and increasing *Lactobacillus* populations in the cecum by 15–22%. The production of SCPs for waste utilization and enzyme production is being researched. *Trichoderma reesei* efficiently utilizes whey as a carbon source and achieves biomass yields of 0.45–0.65 g/g lactose, with protein contents of 45–55% and cultivation productivity of 0.8–1.2 g/L/h in optimized bioreactors. In addition, SCP-producing fungi secrete valuable enzymes such as proteases and cellulases, which can be used for biofuels and increase enzyme activity by 50–75% under optimized fermentation conditions. *T. reesei*, which grows on lignocellulosic substrates, reaches cellulase activities of 1.2–2.8 FPU/mL. Fungal SCPs are analyzed for their bioactive properties. SCP-derived peptides show antimicrobial effects, with *Saccharomyces cerevisiae* protein hydrolysates inhibiting *E. coli* and *Staphylococcus aureus* by 65–80% at 0.5–1.5 mg/mL, with MIC of 0.18–0.42 mg/mL against common foodborne pathogens. In addition, SCP-derived β-glucans improve immune function by increasing macrophage activity by 40–60% and natural killer (NK) cell cytotoxicity by 25–38% at a dosage of 100–250 mg/day, making them promising functional ingredients for immunological supplements. SCP bread enriched with 5–10% mushroom protein increases the protein content by 18–25% while retaining 85% of the sensory acceptability. *Trichoderma reesei* also produces enzymes such as cellulases (1.2–2.8 FPU/mL), with 50–75% higher activity under optimized conditions. SCP-derived peptides show antimicrobial activity and inhibit *E. coli* and *S. aureus* by 65–80% at 0.5–1.5 mg/mL (MIC 0.18–0.42 mg/mL), while β-glucans improve immune function by increasing macrophage activity by 40–60% and NK cell cytotoxicity by 25–38% at 100–250 mg/day. Although SCPs show multifunctional potential in various areas, consistent bioactivity and integration into existing systems require further standardization and validation.	Food industry, feed industry, biotechnology, pharmaceuticals	[237,238,239,240,241,242]

## Data Availability

No new data were created or analyzed in this study. Data sharing is not applicable to this article.
